# Partial Purification and Characterisation of Pectinase Produced by *Aspergillus niger* LFP-1 Grown on Pomelo Peels as a Substrate

**DOI:** 10.21315/tlsr2021.32.1.1

**Published:** 2021-03-31

**Authors:** Mohd Taufiq Mat Jalil, Darah Ibrahim

**Affiliations:** 1 School of Biology, Faculty of Applied Sciences, Universiti Teknologi MARA, 40450 Shah Alam, Selangor, Malaysia; 2 Industrial Biotechnology Research Laboratory (IBRL), School of Biological Sciences, Universiti Sains Malaysia, 11800 USM Pulau Pinang, Malaysia

**Keywords:** Pectinase, Solid State Fermentation, *Aspergillus niger* LFP-1, Purification, Characterisation, Enzim Pektinase, Perfermentasian Keadaan Pepejal, *Aspergillus niger* LFP-1, Penulenan, Pencirian

## Abstract

In the present study, pectinase was produced by local fungal isolate, *Aspergillus niger* LFP-1 grown on pomelo peels as a sole carbon source under solid-state fermentation (SSF). The purification process begins with the concentration of crude enzyme using ammonium sulfate precipitation and followed by purification using anion-exchange column chromatography (DEAE-Sephadex) and subsequently using gel filtration column chromatography (Sephadex G-100). On the other hand, the molecular weight of the purified enzyme was determined through SDS-PAGE. The findings revealed the crude enzyme was purified up to 75.89 folds with a specific activity of 61.54 U/mg and the final yield obtained was 0.01%. The molecular mass of the purified pectinase was 48 kDa. The optimum pH and temperature were 3.5 and 50°C, respectively. This enzyme was stable at a range of pH 3.5 to 4.5 and a relatively high temperature (40°C–50°C) for 100 min. The K_m_ and V_max_ were found to be 3.89 mg/mL and 1701 U/mg, respectively. Meanwhile, pectin from citrus fruit and the metal ion (Co^2+^) were the best substrate and inducer to enhance pectinase yield, respectively.

## Abstract (ms)

Dalam kajian ini, enzim pektinase telah dihasilkan dari kulat pencilan tempatan, *Aspergillus niger* LFP-1 yang tumbuh di atas kulit limau bali sebagai satu-satunya sumber karbon melalui kaedah perfermentasian keadaan pepejal. Proses penulenan ini bermula dengan pemekatan enzim kasar menggunakan kaedah pemendapan ammonium sulfate dan diikuti dengan penulenan menggunakan kolum kromatografi pertukaran-ion (DEAE-Sephadex) dan disertai dengan kolum kromatografi penapisan gel (Sephadex G-100). Selain itu, berat molekul enzim yang telah ditulenkan ditentukan menggunakan SDS-PAGE. Keputusan kajian menunjukkan enzim kasar telah ditulenkan sebanyak 75.89 kali ganda dengan aktiviti spesifik sebanyak 61.54 U/mg dan hasil akhir yang diperolehi sebanyak 0.01%. Berat molekul enzim kasar ini adalah 48 kDa. pH dan suhu yang optima masing-masing adalah 3.5 dan 50°C. Enzim ini didapati stabil pada julat pH 3.5 hingga 4.5 dan julat suhu yang tinggi (40°C–50°C) untuk 100 minit. Nilai K_m_ dan V_max_ yang diperoleh masing-masing adalah 3.89 mg/mL and 1701 U/mg. Sementara itu, pektin daripada buah-buahan sitrus dan ion besi (Co^2+^) masing-masing adalah substrat dan pemangkin terbaik untuk meningkatkan hasil pektinase.

**Highlights**

- Pectinase was successfully produced by local fungal isolate, *Aspergillus niger* LFP-1 grown on pomelo peels as a sole carbon source under solid-state fermentation.
- Crude enzyme was purified up to 75.89 folds with a specific activity of 61.54 U/mg and the final yield obtained was 0.01%.
- Pectin from citrus fruit and the metal ion (Co^2+^) were the best substrate and inducer to enhance pectinase yield, respectively.

## INTRODUCTION

Pectinase is one of the valuable enzymes in the industry especially in fruit juices and beverages, paper making and textile industries. Pectinase is a term used for enzymes that capable of hydrolase pectic substances which include polygalacturonase, pectinesterase, pectin lyase and pectate lyase (Singh *et al.* 1999). Ceci and Lozano (1998) defined pectinase as a group of an enzyme that attack and depolymerise pectin by hydrolysis and de-esterification as well as trans-elimination by hydrolysing the ester bond between the carboxyl and methyl of groups pectin in a particular reaction.

Pectinases can be produced either by submerged fermentation (SmF) (Kapoor *et al.* 2000) or solid-state fermentation (SSF) (Antier *et al.* 1993; Acuna-Arguelles *et al.* 1994). Pomelo is one of the citrus fruits that has thick peels contain a lot of pectins. The pectin fibers which are polysaccharides substances are responsible for adherence of the peels to the fruits (Pretel *et al.* 2008). In fact, in the industry processing citrus fruits, enzymes such as pectinase have been used to separate the peel and fruit. Production of enzyme degrading pectin has a significant role in citrus industries since pulp, peel and membrane of orange and other citrus fruit are highly susceptible to hydrolysis by the mixture of both pectinase and cellulase. Both of enzyme mixture is very crucial to complete the conversion of all carbohydrates to monomeric sugars (Grohmann *et al.* 1994; Grohmann & Baldwin 1992). Since pomelo peel waste contains a lot of pectins and easy to grind, it can be used as a substrate in SSF to produce pectinase by microbes such as fungal, bacteria as well as yeast. The re-use of agro-industrial pomelo peels and pulp waste has a significant good effect in converting the accumulating citrus waste to a beneficial enzyme such as pectin and also can convert the waste to the wealth. Furthermore, the use of pomelo as a substrate for enzyme production can help farmers and citrus industries to dispose of the pomelo peels in an eco-friendly manner and in addition to use the fermented waste as a bio-fertiliser (Attyia & Ashour 2002). Thus, it can be used as an abundant, cost-effective and natural source of pectinase.

The purification and characterisation of the enzyme are crucial since optimal fermentation conditions and enzyme recovery can reduce the production cost (Siddiqui *et al.* 2012). Besides that, the assessment of enzyme kinetics could reveal the nature of the catalytic mechanism role in metabolism, enzyme inhibition and activity control (Meena *et al.* 2015). In the present study, pectinase produced by *Aspergillus niger* using pomelo peel as a substrate via SSF was purified and characterised by accessing the optimal temperature and pH. Meanwhile, the molecular mass of the enzyme was determined as well as Michaelis-Menten constant.

## MATERIALS AND METHODS

### Substrate Preparation

Pomelo peels were obtained from a stall at Changlun, Kedah, Malaysia and the peels were chopped into small pieces of uniform size, spread on the trays and dried under sunlight until a constant weight was obtained. Then it was ground to powder form (particle size = 0.75 mm) prior to its use in SSF using an electrical grinder (NEWTRY 700 g Electric Grinder 2400 W, China).

### Microorganism and Inoculum Preparation

*A. niger* LFP-1 provided from the stock fungal cultures of the Industrial Biotechnology Research Laboratory (IBRL), School of Biological Sciences, Universiti Sains Malaysia, Pulau Pinang, Malaysia was used for pectinase production throughout the study. The fungal culture was maintained on potato dextrose agar (PDA) slant nourished with 1.0% citrus pectin (w/v) at 30°C for 7 days (Darah *et al.* 2013). The fungal isolate was sub-cultured once a month to ensure its purity and viability. The spore suspension was harvested by scraping the surface of sporulating slants in 10 mL of 0.85% sterile saline solution.

### Cultivation and Extraction

Cultivation of fungal culture was performed by introducing 1 mL of inoculum suspension (1 × 10^7^ spores/mL) into 250 mL Erlenmeyer flasks containing 5 g pomelo peels powder (0.75 mm particle size) which previously moistened with 4 mL of a solution containing 1.2% ammonium nitrate and autoclaved at 121°C for 15 min. The flasks were incubated at room temperature for 7 days under static conditions. After a week of cultivation, the cultures were harvested by mixing with 50 mL of distilled water (Pandey *et al*. 1995) and stirred in an Orbital Shaker – Incubator (ES 20/60, BIOSAN, Latvia) at 150 rpm for 30 min. After that, the medium was filtered using Whatman No. 1 paper to separate supernatant and fungal biomass. The supernatant was used to determine enzyme activity and protein concentration in the subsequent experiment (Suresh *et al.* 2009).

### Pectinase Activity

Pectinase assay was carried out using the DNS method (Miller 1959). The reaction mixture (1.0 mL) containing equal amounts of the substrate (0.5%) prepared in citrate buffer (0.05 M pH 4.4) and the suitably diluted enzyme was incubated at 50°C for 30 min in a water bath. After incubation, 3 mL DNS solution was added to stop the reaction and tubes were kept in boiling water for 10 min. On cooling, the developed colour was read at 575 nm using a UV-visible spectrophotometer. The amount of released reducing sugar was quantified using D-galacturonic acid as a standard. The enzyme activity was calculated as the amount of enzyme required to release one micromole equivalent of D-galacturonic acid per minute under assay condition.

### Protein Determination

Protein concentration was determined using Lowry’s method, with bovine albumin serum as the standard (Lowry *et al.* 1951).

### Purification of Pectinase

The procedure used to purify pectinase from *A. niger* LFP-1 involved the following three steps. All of the purification steps were carried out at 4°C to conserve enzyme activity unless stated otherwise.

#### Step 1: Ammonium sulfate precipitation

The supernatant was precipitated with 80% saturation of solid ammonium sulfate at 4°C. Precipitation was allowed to continue overnight, which was followed by centrifugation at 3000 ppm for 30 min (Buga *et al.* 2010). The precipitate was then dissolved in 0.05 M citrate buffer (pH 4.4) and any excess salt was removed using an Econo-Pac 10 DG desalting column (Bio-Rad). The filtrate was then concentrated using centrifugal ultrafiltration (Vivaspin 20, 5 kDa cut-off, Sartorius) and used as starting material for the purification process.

#### Step 2: Anion-exchange chromatography

The concentrated crude enzyme was subjected to anion exchange chromatography (19 cm × 0.7 cm) using a column of DEAE Sephadex (Sigma-Aldrich, St. Louis, Missouri). An amount of 0.4 mL of the concentrated enzyme was loaded to DEAE-Sephadex (Sigma-Aldrich, St. Louis, Missouri) which was previously washed and equilibrated with 0.05 M citrate buffer (pH 4.4). After equilibration, the enzyme was eluted with the same buffer and 3 mL of the sample was then collected. The active fraction was then pooled and collected together for the subsequent purification step.

#### Step 3: Gel filtration chromatography

Subsequently, the active fractions from the previous step were loaded to the top of the Sephadex-100 column which was pre-equilibrated 0.05 M citrate buffer (pH 4.4). The fractions were eluted with the same buffer at the flow rate of 0.41 mL/min. The fractions of each purification steps were assayed for enzyme activity.

### Molecular Weight Determination (Zymogram Analysis)

The molecular weight of purified pectinase was determined by sodium dodecyl sulfate-polyacrylamide gel electrophoresis (SDS-PAGE, BIO-RAD, California, United States). The enzyme was treated with sample buffer (125 mM Tris-HCl buffer, pH 6.8, 4% SDS, 10% (v/v) Glycerin, 10% (v/v) (2-Mercaptoethanol) and incubated at 95°C for 5 min in a Thermomixer Incubator (TSI-100, Biobase, China). After running, the gel was stained with 0.25% Bromophenol blue followed by de-staining and drying. The authentic broad range protein molecular weight markers (Promega Corporation, Madison, Wisconsin, United States) contains standards molecular weight of 10 kDa, 15 kDa, 35kDa, 50 kDa, 75kDa, 100 kDa, 150 kDa, and 225 kDa was used.

### Characterisation of Pectinase

#### Effect of temperature and thermal stability

The effect of incubation temperature (25°C, 30°C, 35°C, 40°C, 45°C, 50°C, 55°C, 60°C, 65°C, 70°C, 75°C, 80°C) on pectinase activity was determined by incubation of the reaction mixture in 50 mM, Pectin-citrate buffer (pH 4.4) for 30 min at each temperature. Meanwhile, the thermal stability of the purified pectinase was evaluated at various incubation temperature degrees (25°C, 30°C, 35°C, 40°C, 45°C, 50°C, 55°C, 60°C, 65°C, 70°C, 75°C, 80°C) from 10 to 100 min (10 min, 20 min, 30 min, 40 min, 50 min, 60 min, 70 min, 80 min, 90 min, 100 min). The residual activity was assayed by the standard method.

#### Effect of pH and pH stability

The effect of reaction pH on pectinase activity was accessed using citrate buffer (pH 2.0–4.0), potassium-phosphate buffer (pH 5.0–8.0) and glycine-NaOH buffer (pH 9.0). The reaction mixture was incubated for 30 min at 50°C. The pH stability of the purified pectinase was determined by pre-incubation of the enzyme at various pH range (pH 2.0–9.0) for 100 min. The residual activity was measured by the standard method.

#### Substrate specificity

The substrate specificity of purified pectinase towards various substrates including xylan, citrus pectin, potato dextrose agar (dextrose), D-galacturonic acid, and starch was evaluated. The substrates were incubated with the purified enzyme in 50 mM citrate buffer (pH 4.4) for 30 min at an optimal temperature. The relative activity of pectinase was determined for each substrate using the standard methods in which pectin from citrus was set as a control.

#### Effect of activators and inhibitors

The influence of chemical compounds on the activity of *A. niger* pectinase was evaluated by incubation of the purified enzyme with 20 mM metal ions and reagents including sodium dodecyl sulphate (SDS), ethylenediamine tetraacetic acid (EDTA), NaCl, KCl, CaCl_2_.2H_2_O, FeSO_4_, FeCl_3_, MgSO_4_ and AgNO_3_. The residual activity was determined using the standard method under optimum conditions.

#### Determination of the Michaelis-Menten constant

The K_m_ and V_max_ values of the enzyme were assessed by measuring the reaction velocity at different substrate concentration and the citrus pectin was used as a substrate. A stock solution of citrus pectin with a concentration of 10 mg/mL was firstly prepared in citrate buffer (pH 4.5). The stock was subsequently diluted by a volume of citrate buffer to make a serial dilution with the final concentration of citrus pectin in between 2 mg/mL to 10 mg/mL. The pectinase activity was determined using the previously standard assay. The relationship between velocity (enzyme activity) and substrate concentration was plotted using GraphPad Prism 8 software. The Michaelis-Menten constant values (K_m_ and V_max_) were calculated using non-regression linear.

### Statistical Analysis

One-way analysis of variance (ANOVA) and Duncan Multiple Range Test (DMRT) with PASW Statistics 18 version has been used to analyse the significant difference of the mean of experimental data. Five percent (5%) confidence level or α = 0.05 has been used to test all experimental data. All measurements were determined in triplicates.

## RESULTS

### Fungal Pectinase Producer

**Figure 1:**
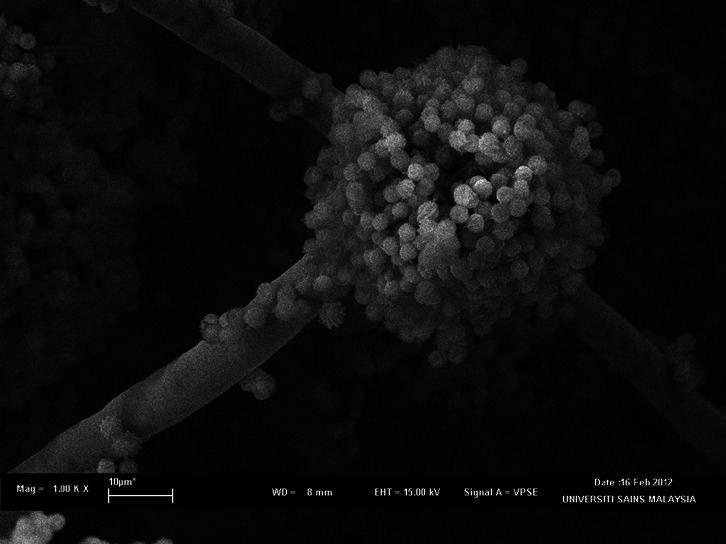

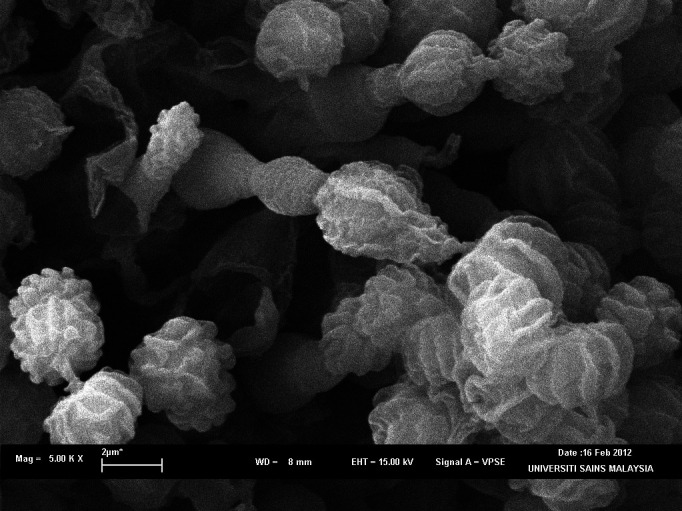
SEM photomicrograph of *A. niger* LFP-1. (a) The *Aspergillus* head with conidiophores (magnification 1.00 KX); (b) The spherical conidia and phialides of the fungal isolate at higher magnification (magnification 5.00 KX).

**Figure 2:**
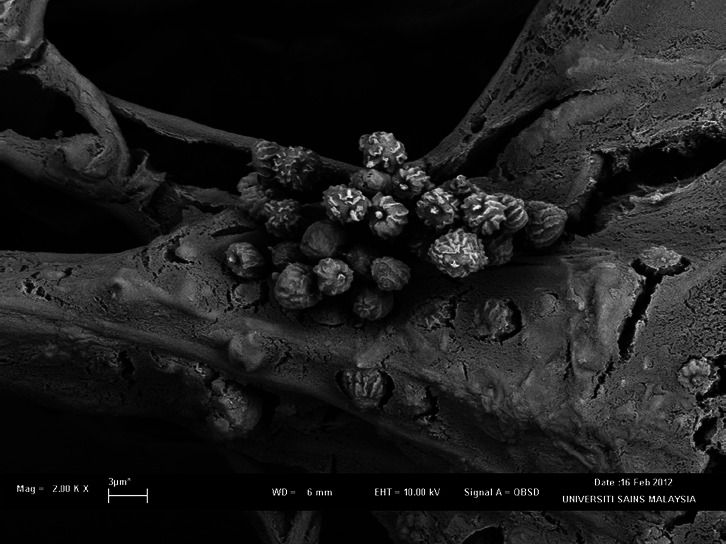

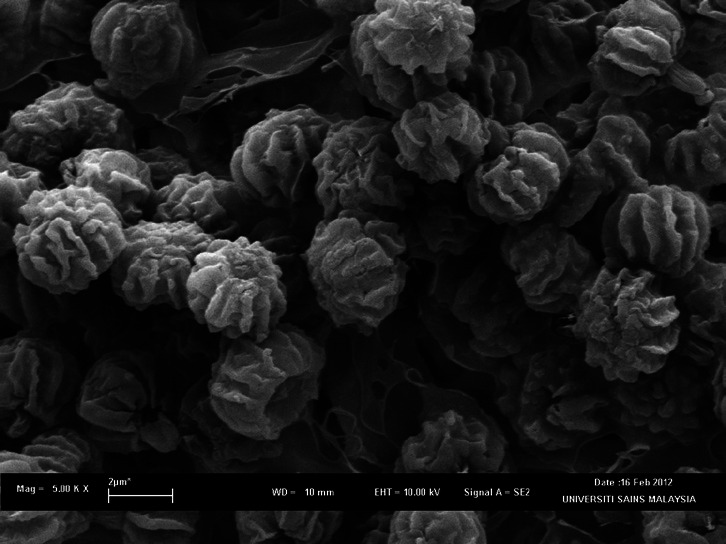
SEM photomicrograph of *A. niger* LFP-1 on substrate. (a) Spore of *A. niger* colonised the substrate (magnification 1.00 KX); (b) The colonisation of fungal isolate on pomelo peels and degradation of substrates at higher magnification (magnification 5.00 KX).

Fungal strain, *A. niger* LFP-1 was previously isolated from rotten orange nearby Industrial Biotechnology Research Laboratory (IBRL) building, Universiti Sains Malaysia, Pulau Pinang is a filamentous fungus which forms filaments and resembles the structure of a plant. Fig. 1(a) shows the SEM photomicrographs of *A. niger* LFP-1 with smooth and colourless conidiophores and spores, whereas Fig. 1(b) illustrates the spherical conidia and phialides of the fungal isolate at higher magnification (5.00 KX). Meanwhile, Fig. 2 shows the SEM photomicrograph of fungal isolate on pomelo peels (substrate). Fig. 2(a) demonstrates the spores of *A. niger* LFP-1 colonising and penetrating the substrates. Fig. 2(b) illustrates the colonisation of fungal isolate on pomelo peels and degradation of substrates at higher magnification (5.00 KX).

### Purification of Pectinase

Crude pectinase was harvested from the filamentous fungus, *A. niger* LFP-1 that utilising pomelo peels as a substrate under SSF. Table 1 summarises the data on the purification of pectinase at various purification steps. The specific enzyme activity was increased up to 14-folds (specific activity: 11.41 U/mg) with 86.51% of yield by ammonium sulfate precipitation step. The highest pectinase activity was detected at fraction 5 with 0.28 U/mL in anion-exchange chromatography. The specific enzyme activity was slightly increased to 16.50 U/mg (20.37 folds) compared to crude enzyme preparation with 0.05% of yield. The partially purified pectinase was then chromatographed on Sephadex G-100 gel filtration. A single peak of pectinase activity was detected at fraction 7 with 0.083 U/mL. After purification steps, the specific enzyme activity was shot up to 61.54 U/mg equivalent to 75.98 folds with 0.01% recovery.

### Zymogram Analysis

**Figure 3:**
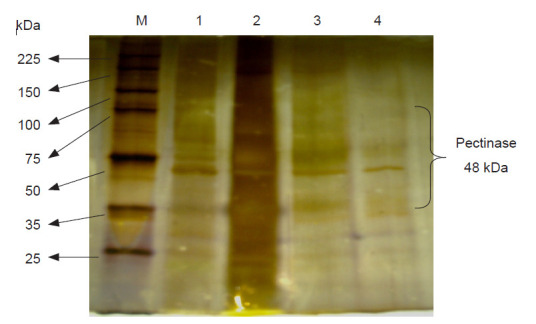
Zymogram analysis of crude, concentrated, partially purified and purified pectinase with 12.5% polyacrylamide (M = unstained protein marker; Lane 1 = crude enzyme; Lane 2 = concentrated enzyme [ammonium sulphate precipitation]; Lane 3 = partial purified pectinase [anion exchange chromatography]; Lane 4 = purified pectinase [gel filtration chromatography]).

**Figure 4:**
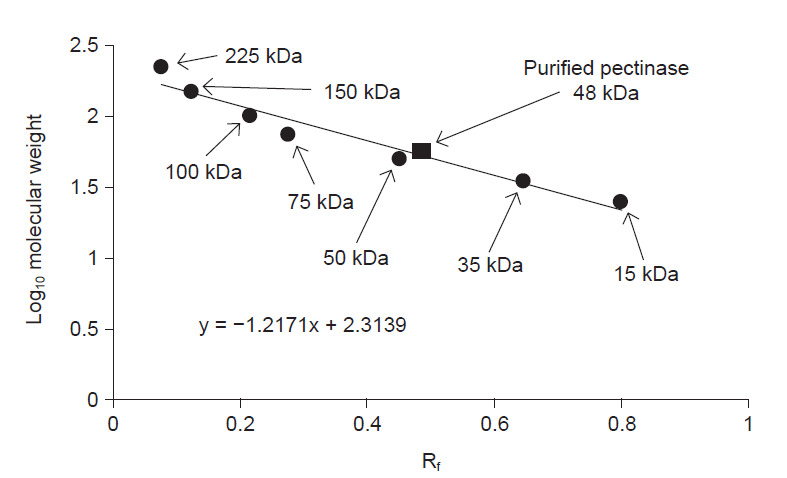
Determination of purified pectinase molecular weight using SDS-PAGE. R_f_ = relative mobility.

The molecular weight of the purified pectinase of *A. niger* LFP-1 was determined under denaturing conditions using SDS-PAGE (Zymogram analysis). Fig. 3 shows the SDS-PAGE analysis of pectinase exhibited a single-band with the molecular mass in between 35 to 50 kDa. A graph of log_10_ molecular weight versus relative mobility (R_f_) was plotted to determine the more accurate molecular mass of purified pectinase and Fig. 4 illustrates the molecular weight of purified pectinase was estimated to be 48 kDa.

### Biochemical Properties of Pectinase

#### Effect of incubation temperature and thermal stability

**Figure 5:**
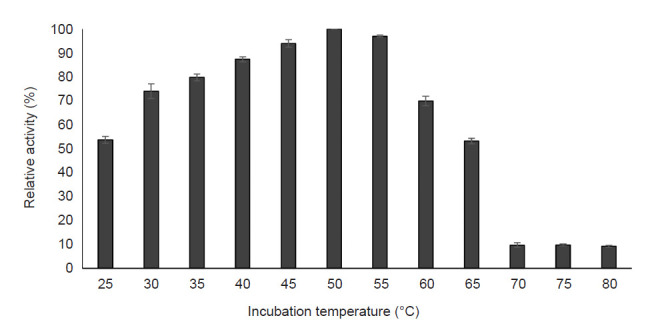
Effect of incubation temperature on pectinase activity.

**Figure 6:**
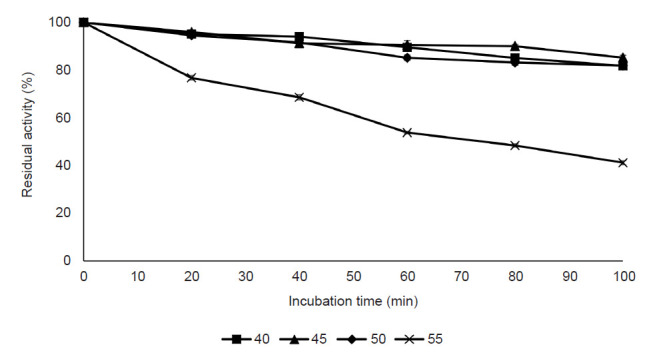
Effect of thermostability on purified pectinase activity.

Fig. 5 shows the effect of reaction temperature on pectinase activity. The pectinase activity was detected in the temperature range 25°C to 80°C, with the optimum temperature of 50°C, followed by 55°C and 45°C. The finding demonstrated that the pectinase activity increased with the increment of temperature until the optimum temperature was achieved. Meanwhile, the pectinase activity dropped sharply at a temperature above 70°C with only 9% of activity left at 80°C. Four temperatures (40°C, 45°C, 50°C and 55°C) with the highest pectinase activity were chosen for thermostability study. Fig. 6 shows the effect of thermal stability on pectinase activity. The result showed that the activity was slightly dropped for 40°C, 45°C and 50°C with more than 80% of activity was retained after 100 min of the incubation period. For 55°C, the enzyme activity was drastically dropped after 20 min of the incubation period (76.73% of activity) and the decrement of activity has continuously occurred with only 41.17% of activity left after 100 min of incubation.

#### Effect of pH and pH stability

**Figure 7:**
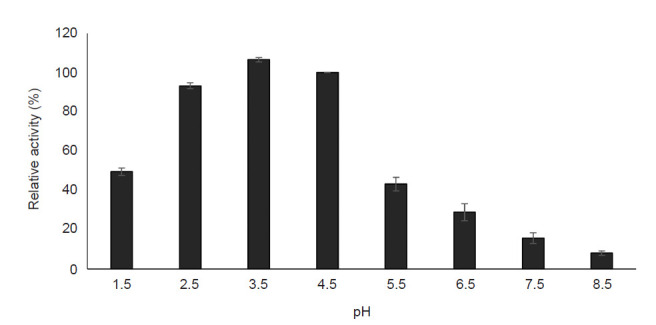
Effect of pH on purified pectinase activity.

**Figure 8:**
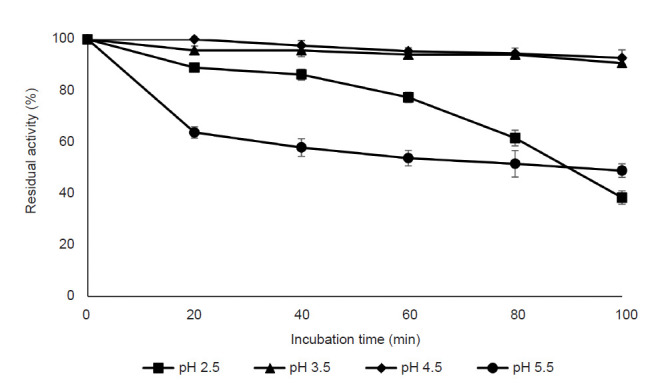
Effect of pH stability on purified pectinase activity.

The influence of pH value (pH 1.5–8.5) on purified pectinase activity was determined in the study. Fig. 7 demonstrates that the optimum pH value for the highest activity was 3.5 in which the pectinase activity reached its maximal value (106.55 ± 1.12%). The lowest pectinase activity was obtained at the alkaline range whereby the enzyme lost about 92% of its activity at pH 8.5. The pH stability of purified pectinase produced by *A. niger* LFP-1 was accessed using a pH range of 2.5 to 5.5 and the result is shown in Fig. 8. The result revealed that the enzyme activity decreased with an increasing pH value of the incubation medium. The purified pectinase retained 90% of its original activity for 100 min at pH 3.5 and 4.5. However, the extremely low pH (pH 2.5) and pH beyond the optimum pH value (pH 5.5) leads to a decrement of pectinase activity with the relative residual drops to 38% and 48%, respectively.

#### Substrates specificity

**Figure 9:**
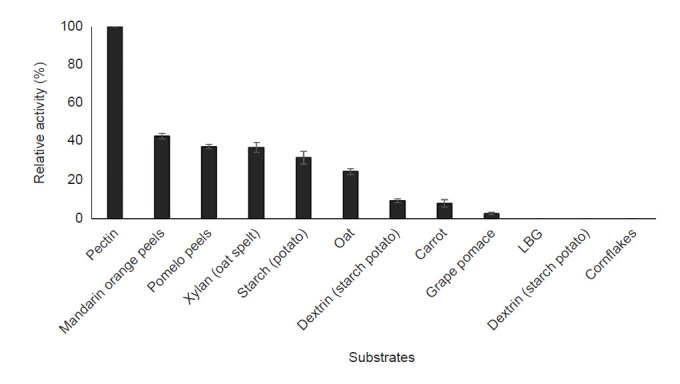
Effect of various substrates on purified pectinase activity.

The affinity of purified pectinase towards substrates was evaluated as shown in Fig. 9. The enzyme has a favourable activity against a fruit-based substrate such as mandarin orange, pomelo peels and hemicellulose substance (xylan) with 42.97 ± 1.4%, 3.752 ± 1.1% and 37.09 ± 2.6% of relative activity, respectively. Besides that, the enzyme was able to degrade polymeric carbohydrate (starch) and oat with up to 20% of relative activity. However, the pectinase was inactive against several substrates including locust bean gum (LBG), dextrin and cornflakes.

#### Effect of metal ion and reagents on pectinase activity

The impact of various metal ions and reagents on the pectinase activity produced by *A. niger* LFP-1 is summarised in Table 2. The enzyme has reached its maximum activity by pretreating with Co^2+^ in which, the relative activity was shot up to 190.13 ± 0.3% compared to the control. Besides, the pectinase was enhanced by several metal ions and reagent with higher activity achieved when the enzyme was pre-incubated with Fe^3+^ (125.04 ± 0.8%), Ca^2+^ (116.02 ± 1.1%), Na^+^ (103.42 ± 2.2%), NH^4+^ (103.42 ± 1.2%), EDTA (115.39 ± 0.2%) and 2-Mercaptoethanol (114.26 ± 1.6%). On the other hand, the enzyme activity was significantly reduced by the presence of Mn^2+^, Al^3+^, Ba^+^ retaining only about 38.19 ± 1.1%, 28.47 ± 2.1% and 6.58 ± 1.2%, respectively. It is noteworthy that Ag^+^ and SDS were completely inhibited pectinase activity whereby no activity was observed.

### Kinetics Study of Pectinase

**Figure 10:**
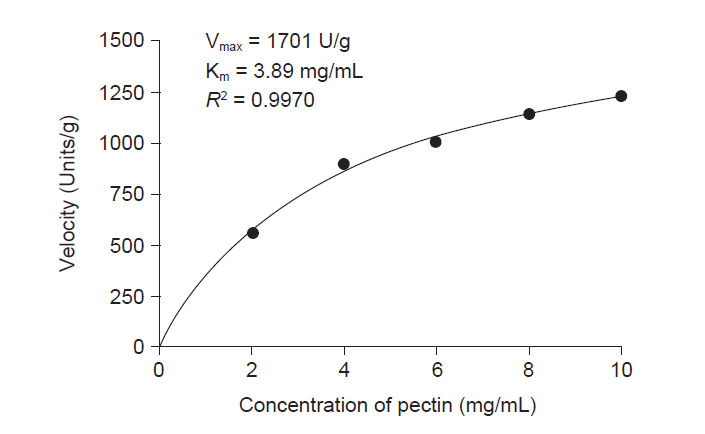
Michaelis-Menten kinetics.

The Michaelis-Menten constant, K_m_ and V_max_ values of pectinase were assessed by measuring the reaction velocity at different concentration of the citrus pectin. The relation between velocity and the substrate concentration was analysed with non-regression analysis. The results showed the regression coefficient (*R*^2^) was 0.9970 which indicated the concentration of the substrate and the enzyme activity was positively correlated (Fig. 10). Furthermore, the regression analysis demonstrated that the K_m_ and V_max_ values of the enzyme were 3.89 mg/mL and 1701 U/mg, respectively.

## DISCUSSION

*A. niger* is a filamentous fungus that widely distributed around the world with the capability to develop in a vast variety of substrates. The species can cause deterioration of food although some of them are used in many fields including the fermentation industry to produce hydrolytic enzymes (amylases and lipases) and organic acids such as citric and gluconic acids (Silva *et al.* 2011). Moreover, the filamentous fungi such as *Aspergillus* sp. have been extensively used in the biotechnological process as cell factories due to their metabolic versatility and capability to secrete high levels of antibiotics, polysaccharides, enzymes, vitamins, and their mycelia also suitable for application in the treatment of textile wastewater and dye (Colin *et al.* 2013).

The fungal strain used in the present study was previously isolated from orange peels and the peels are favourable to the growth of *A. niger* since it contains soluble sugars and pectin as the main components. According to Rivas *et al.* (2008), the orange peels consist of fiber (pectins: 42.5%, hemicelluloses: 10.5%, cellulose: 9.21%, lignin: 0.84%), soluble sugars (16.9%), protein (6.5%), starch (3.75%), ashes (3.5%) and fats (1.95%). However, the total sugar content may vary from 29% to 44% (Torrado et *al.* 2011). Grewal and Kalra (1995) reported the strains of *A. niger* need a fair higher sugar concentration in the medium to support its growth and it has been reported to utilise various types of substrates for enzyme production through SSF. For instance, Abbas *et al.* (2011) reported that the fungal strain, *A. niger* could produce polygalacturonase via SSF by utilising a mixture of apple bagasse and wheat bran as a substrate. They revealed that the highest pectinase activity achieved after 8 days of cultivation at 30°C with the relative humidity of the cultivation medium was 70%. Meanwhile, another previous study reported that *A. niger* able to produce pectinase by utilising pomelo peels as economic alternative substrates (Darah *et al.* 2013) and optimisation of cultural conditions through swallow tray system could enhance enzyme production up to 50% (Darah *et al.* 2015).

The purification of protein and the desired enzyme is crucial for designing and selection of pectinase. Moreover, the improved knowledge regarding biochemical properties of microbial pectinase is essential to have a better understanding of pectinase mechanism of action, to commercialise the enzyme as well as to study the uses of the enzyme in various potential fields (Zhang *et al.* 2009). The purification of pectinase has several series of methods such as salt/solvent precipitation, gel filtration chromatography, ion-exchange chromatography (Lekha & Lonsane 1991) and followed by determination of the molecular weight of pectinase using SDS-PAGE. Ammonium sulfate precipitation was performed in the present study as an initial step to purify extracellular pectinase from *A. niger* LFP-1. Ammonium sulfate at a concentration level of 80% was used after the preliminary study was done. A high concentration of ammonium sulfate was used since the salting-out process dependent on the hydrophobicity of the protein and thus, high salt concentration promotes the aggregation of hydrophobic patches on the protein surface (Farinas *et al.* 2011). Furthermore, the addition of salt in high concentration reduces the electrostatic repulsion between like-charged groups at the protein surface and disturbs the structure of water molecules around the protein, making the aqueous salt solution a poor solvent for proteins, which precipitate out (Kumar *et al.* 2003). Many researchers implemented the precipitation method as the first step in their purification process. For instance, Ahmed *et al.* (2016) reported that 23.6 U/mg of specific activity with 120 mg/mL protein was obtained after purification of pectinase from *A. niger* grown on citrus waste peels through ammonium sulfate precipitation. Besides, the method has been used to partially purify crude pectin lyase by Batool *et al.* (2013) and they reported that the maximum enzyme activity (382.45 U/mL/min) was achieved at 60% ammonium sulfate concentration.

Ion-exchange chromatography is a common method used for protein purification and it relies on the charge-to-charge interactions between the charges immobilised on the ion-exchange resins and the proteins in the lysate (Adhikari *et al.* 2010). The method provides high resolution even though under mild conditions with high binding capacity (Saraswat *et al.* 2013). There are two types of ion-exchange chromatography which are cation and anion-exchange although the anion-exchange was often used in tandem for affecting removal of impurities including DNA (deoxyribonucleic acid) and LPS (lipopolysaccharides) for the feed (Yang *et al.* 2008). As for the present study, the pectinase from *A. niger* LFP-1 was purified up to 20.30 folds through DEAE-cellulose anion-exchange chromatography (Table 1). Patidar *et al.* (2017) performed the chromatography on DEAE-cellulose column (2 cm × 15 cm) to purify polygalacturonase enzyme from *A. niger* AN07 cultured on dried papaya peels and the enzyme was purified to 24.8 folds with a 52.6% recovery. Meanwhile, pectinase produced by *A. fumigatus* has been purified 1.74 folds which equivalent to 15.19 U/mg of specific activity after purification process through CM Sephadex C-50 ion-exchange chromatography (Okonji *et al.* 2019). However, the present study (Table 1) revealed the enzyme yield was significantly decreased upon anion-exchange chromatography and this may be due to the selection of buffer, pH of the buffer, protein loss and impurities in the enzyme. This assumption supported by Nooralabettu (2014) who revealed that the efficiency of anion-exchange chromatography depends on suitable binding and elution buffer, ionic strength, flow rate and optimum pH.

Gel filtration chromatography or sometimes referred to as size exclusion chromatography is a chromatography technique that allows the separation of macromolecules based on their hydrodynamic size whereby the larger molecules can only penetrate larger pores (elute earlier) whereas the smaller molecules can access the higher number of pores and retain longer in the column (Raak *et al.* 2018). The present study revealed that the pectinase was purified up to 76 folds with a specific activity of 61.54 U/mg through gel filtration chromatography (Table 1). However, the purification folds and specific activity of enzyme purified by gel filtration chromatography may vary depends on the microbial strains and substrates. Ire and Vinking (2016) reported that the polygalacturonase from *A. niger* grown on banana peels was purified up to 42 folds with 166.67 U/mg of specific activity trough gel filtration on Sephadex 100. On the other hand, the pectinase from *A. niger* strain MCAS2 was purified 8.5-folds with a specific activity of 60 U/mg by using Sephadex G-75 gel filtration chromatography (Khatri *et al.* 2015). Overall, the purification steps demonstrate a decrease in total activity and total protein. This phenomenon might be due to the removal of impurities from the crude which is responsible for high enzyme activity and total protein (Ire & Vinking 2016). However, the purification process enhanced specific pectinase activity. Similar observation reported by Ubani *et al.* (2015) who revealed that the purification steps increased specific pectinase activity from crude (76.04 U/mg) to the purified enzyme (179.18 U/mg). They also postulated that as purification steps increased, the enzyme activity also increased.

The present study revealed that the molecular weight of pectinase from *A. niger* LFP-1 grown in pomelo peels as a substrate and sole carbon source was approximately 48 kDa. Similar observation reported by Trindade *et al.* (2016) who revealed the molecular mass of pectinase from thermophilic fungus, *Rhizomucor pusillus* A13.36 was estimated to be in the range of 43.5 kDa–47 kDa. The finding was in agreement with the previous study that reported the molecular weight of microbial pectinases to fall within the size in the range of 30 kDa–70 kDa (Okonji *et al.* 2019). Alkaline thermostable pectinase purified from *A. niger* strain MCAS2 showed the molecular weight of 66 kDa (Khatri *et al.* 2015). Furthermore, pectinase isolated from *A. niger* cultured in citrus waste peels demonstrated a molecular weight of 30 kDa. However, the molecular weight of microbial pectinases may vary depends on microbial strains, substrates, and types of the fermentation process. For instance, Kashyap *et al.* (2000) reported that the purified pectinase produced by *Bacillus* sp. DT7 was found to have a molecular mass of 106 kDa. Besides that, bacterial pectinase from *Bacillus paralicheniformis* CBS32 cultivated under SmF in shake flask system showed a molecular weight of 110 kDa (Saifur Rahman *et al.* 2019).

The effect of cultivation temperature on enzyme activity demonstrated that 50°C was the optimum temperature for pectinase activity, though there is an appreciable enzyme activity (90%–95% of relative activity) observed at 45°C and 55°C (Fig. 5). However, a decline in the enzyme activity with a temperature beyond the optimum temperature observed. Similar observation reported by Ahmed *et al.* (2016) who revealed that 50°C was the optimum temperature of purified pectinase from *A. niger*, whereas the enzyme activity was suppressed beyond the optimal temperature. According to Meena *et al.* (2015), denaturation of the enzyme occurs in elevated temperature and thus, rapidly reduces the enzyme activity along with the increased temperature. On the other hand, the pectinase from *A. niger* LFP-1 was more stable at a temperature below its optimum temperature, which is 40°C, 45°C and 50°C. The results are in accordance with those reported by Maciel *et al.* (2011), studying pectinase from *A. niger* URM4645 grown on forage palm in SSF. They revealed that the enzyme activity was stable at 50°C and deactivated at higher temperature. The unstable enzyme activity at higher temperature might be due to hydrolysis of peptide bonds, destruction of disulfide bonds, deamination and oxidation of the amino acid side chains of protein molecules (Ire & Vinking 2016).

pH plays a crucial role in pectinase production since it promotes and regulates the synthesis of the extracellular enzyme by microorganisms particularly fungi (Abdullah *et al.* 2018). According to Kulkarni *et al.* (2007), pH was greatly influenced the enzyme activity as the binding of substrate and catalyst are affected by the charge distribution on enzyme molecules and substrate. The present study revealed that pH 3.5 was the optimum pH for pectinase activity (106.55% of relative activity) produced by *A. niger* LFP-1 while pH 4.5 showed appreciable enzyme activity (100% of relative activity, Fig. 7). The finding was in agreement with Ahmed and Sohail (2020) who found that the pectinase was more active towards acidic pH in the range of 3 to 5.5. On the other hand, the studied pectinase was stable at pH in the range of 3.5 to 4.5. Similar observation reported by Silva *et al.* (2002), studying the pectinase from *Penicillium viridicatum* RFC3 grown on agriculture waste with maximal stability achieved at pH 3.5–4.5. According to Robinson (2015), the enzyme should be in optimum pH at which the velocity of the catalysed reaction is maximal and if the pH below or beyond the optimum value, the velocity is declined.

The effect of substrate specificity was evaluated, and the finding revealed the pectin was the major substrate for pectinase enzyme. This finding was consistent with Chowdhury *et al.* (2017), studying pectinase secreted by *Rhizopus oryzae*. Furthermore, orange peels, pomelo peels and xylan demonstrated a favourable substrate for pectinase. The previous study reported that natural substrate, orange and xylan supported the pectinase production with 363.0 ± 2.1 U/g and 697.23 ± 11.7 U/g pectinase activity achieved, respectively (Phutela *et al.* 2005; Oumer & Abate 2017). However, several substrates for pectinase’s substrate specificity have been used in the previous studies. Siddiqui *et al.* (2012) reported polygalacturonase acid has been used as a substrate in the substrate specificity study with 8.34 U/mL of pectinase activity (100% relative activity) was obtained. Besides, another study used digalacturonic acid as a substrate in the enzyme specificity study for pectinase of *Streptomyces lydicus* with 56% of enzyme activity achieved (Jacob *et al.* 2008).

The purified *A. niger* LFP-1 pectinase was subjected to different metal ions and reagents to evaluate their activation and inhibition effects on its activity. The finding revealed that metal ions such as Co^2+^, Fe^3+^, Ca^2+^, Na^+^ and NH^4+^ significantly enhanced pectinase activity with Co^2+^ showed the highest activity. The finding was consistent with Karbalaei-Heidari and Rastegari (2014), characterising the thermostable pectinase from *Bacillus* sp. strain BR1390. Similarly, Ire and Vinking (2016) reported that Co^2+^ strongly stimulated pectinase activity by *A. niger* with 126% relative activity. The activation effect might be due to charge neutralisation on the pectin polymer and thus, reduce the repulsion between the overall negative charge of the enzyme and the pectin (Alkorta *et al.* 1998). On the other hand, metal ion (Ag^+^) was strongly inhibited the pectinase activity. Similar observation reported by Anand *et al.* (2017) who revealed that the Ag^+^ was completely inhibited exo-polygalacturonase activity produced by *A. niger* MTCC 478. The formation of homogalacturonan cross-link by a cation such as Mg^2+^ decreased the availability of the substrate to the enzyme or the binding of cation to an acid amino side chain involved in catalysis of the substrate might be the reason on the inactivation effect of the metal ion on enzyme activity (Okonji *et al.* 2019). Besides that, the surfactants such as EDTA and 2-Mercaptoethanol enhanced enzyme activity with moderate stimulatory whereas the SDS was completely inhibited the enzyme activity. A non-ionic surfactant such as EDTA is believed to improve the activity of a surface-active enzyme in cationic W/O microemulsion by increasing the flexibility of interface and reducing the surface charge density (Shome *et al.* 2007). Similar observation reported by Oumer and Abate (2017) revealed EDTA enhanced pectinase activity with relative activity of 165.3% whereas the presence of SDS in the enzyme-substrate system significantly suppressed the enzyme activity.

The kinetics parameter is crucial in enzymatic reaction since it describes the efficiency of the enzyme. The present study demonstrated that the pectinase from *A. niger* LFP-1 has a relatively highest affinity towards the substrate due to its lowest K_m_ value. Furthermore, the enzyme has the highest utility of pectin (substrate) as a result of its highest V_max_. The finding revealed that a small quantity of the enzyme will digest a considerably high amount of substrate.

## CONCLUSION

Pectinase from *A. niger* LFP-1 which was isolated from orange peels was successfully purified. The biochemical properties demonstrated by the pectinase enzyme suggested that this particular enzyme is a potential candidate for various biochemical reactions in the industrial biotechnological process, especially in the juice and food industry.

## Figures and Tables

**Table 1:** Summary of purification profile of pectinase from *A. niger* LFP-1.

Purification steps	Total activity (U)	Total protein (mg)	Specific activity (U/mg)	Purification (Fold)	Yield (%)
Pectinase crude enzyme	2275.00	2800.00	0.81	1.00	100
Ammonium sulphate precipitation	1968.00	172.50	11.41	14.09	86.51
Anion exchange chromatography	0.99	0.06	16.50	20.37	0.05
Gel filtration chromatography	0.24	0.0039	61.54	75.98	0.01
